# Targeting the ERK2 Signaling Pathway With Frog Skin‐Derived Bioactive Peptides: Insights From Molecular Modeling, Molecular Dynamics, and Physicochemical Features

**DOI:** 10.1002/open.70295

**Published:** 2026-08-24

**Authors:** Nahlah Makki Almansour, Mahmoud M. H. Abdelhamid, Zahraa Alali, Mahmoud A. A. Ibrahim

**Affiliations:** ^1^ Department of Biology College of Science University of Hafr Al Batin Hafar Al Batin Saudi Arabia; ^2^ Computational Chemistry Laboratory Chemistry Department Faculty of Science Minia University Minia Egypt; ^3^ Department of Clinical Laboratory Sciences College of Applied Medical Sciences University of Hafr Al Batin Hafar Al Batin Saudi Arabia; ^4^ Department of Supportive Requirements University of Technology and Applied Sciences Nizwa Sultanate of Oman; ^5^ School of Health Sciences University of KwaZulu‐Natal Durban South Africa

**Keywords:** ERK2, frog skin‐derived peptides, molecular dynamics, molecular modeling, physicochemical properties

## Abstract

ERK2 plays a vital function in promoting cell survival and proliferation by phosphorylating various substrates. ERK2 is a key druggable target owing to its function in uncontrolled cell proliferation and cancer, making selective ERK2 inhibitors essential for treating aggressive malignancies. Peptides are promising therapeutic agents with strong biological activity, and frog skin‐derived peptides have shown notable anticancer potential. Seventeen curated frog skin‐derived anticancer peptides were modeled and docked against the ERK2 D‐recruitment site (DRS) using HADDOCK2.4 software. The predicted peptide‐DRS complexes were introduced to molecular dynamics simulations (MDS), and their binding energies (Δ*G*_binding_) were evaluated utilizing the MM/GBSA approach. Upon MM/GBSA//200 ns MDS, dermaseptin‐B3, dermaseptin‐PD‐1, and ascaphin‐8 showed better binding affinities against ERK2 DRS, with Δ*G*_binding_ values of −77.8, −74.1, and −56.7 kcal/mol, respectively, compared with the structurally resolved DRS‐binding reference peptide KIM‐MAP3 (Δ*G*_binding_ = −51.5 kcal/mol). Post‐MD analyses, including binding energy per trajectory and RMSD analyses, affirmed the great stability of the identified peptides complexed with ERK2 DRS over 200 ns MDS. The predicted physicochemical features of the identified peptides indicated favorable functional potential. Collectively, these computational outcomes highlighted dermaseptin‐B3, dermaseptin‐PD‐1, and ascaphin‐8 as promising ERK2 DRS‐binding candidates. Further experimental validation is required to confirm their functional inhibitory activity.

## Graphical abstract

Frog skin‐derived peptides are screened for prospective ERK2 inhibitors using docking calculations, molecular dynamics, MM/GBSA binding energy calculations, and physicochemical properties. The results propose that dermaseptin‐B3, dermaseptin‐PD‐1, and ascaphin‐8 can act as effective inhibitors of ERK2, warranting further in‐depth in vitro and in vivo investigations.

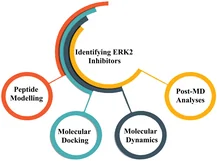

## 1 Introduction

Mitogen‐activated protein kinases (MAPKs) are a highly conserved class of protein kinases that play a fundamental function in regulating intracellular signaling pathways in eukaryotic cells [1, 2, 3, 4]. MAPK signaling pathways are triggered by a variety of extracellular cues and modulate numerous downstream effectors through phosphorylation. MAPK pathways regulate cellular processes, such as metabolism, proliferation, and survival. Various tumors and other pathological circumstances have been closely linked to the onset and progression of dysregulated MAPK signaling [5]. The extracellular signal‐regulated kinase (ERK), p38 kinase, and c‐Jun N‐terminal kinase (JNK) pathways are the most extensively characterized of the MAPK cascades [6].

**FIGURE 1:**
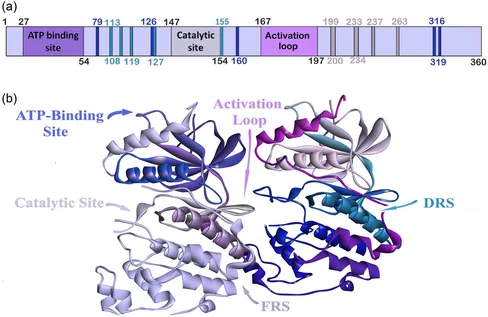
(a) 2D sequence representation with main domains and (b) 3D structure of ERK2, including activation loop, ATP‐binding site, FRS, catalytic site, and DRS. The DRS is displayed as a noncatalytic docking interface involved in the recruitment of ERK2 substrates and regulators.

The ERK1/2 are the most distal kinases in the canonical MAPK/ERK signaling cascade. ERK1/2 are triggered by a wide array of extracellular stimuli, such as cytokines and growth factors, and subsequently control diverse cellular processes, involving differentiation, cell proliferation, and survival [7, 8]. Among them, ERK2 has a central role in downstream signal transduction by phosphorylating multiple transcription factors, involving ELK1, c‐Fos, and p53, which are key regulators of cell proliferation and oncogenic transformation [9]. Structurally, ERK2 comprises several key functional regions, including the activation loop, catalytic site, ATP‐binding site, and docking sites (Figure 1) [10, 11, 12]. The activation loop extends from ASP167 to GLU197 and includes the conserved residues THR185 and TYR187; dual phosphorylation of this segment by upstream MEK kinases triggers conformational rearrangements that switch ERK2 from an inactive to an active state [10]. The catalytic site includes the conserved HRDLKPEN motif within the catalytic loop, with ASP147 acting as a critical residue for phosphoryl transfer during substrate phosphorylation [10]. The ERK2 ATP‐binding site comprises the glycine‐rich loop (GLY32‐GLY37) and the conserved LYS54 residue, which collectively engage the phosphate moieties of ATP. In addition, the *β*1‐ and *β*2‐strands, combined with VAL39, contribute to interactions with the adenine moiety of ATP [10]. MAPKs, including ERK2, also possess two distinct docking regions: the F‐recruitment site (FRS) and the D‐recruitment site (DRS) [10, 11, 12]. These regions govern kinase selectivity, properly align the substrate phosphorylation site with the catalytic core, and increase the local concentration of interacting partners, involving substrates, kinases, scaffold proteins, and phosphatases [12, 13, 14]. Although Figure 1 highlights the major functional regions of ERK2, a detailed comparison of inactive and active ERK2 conformations was not included, as the present study focuses on peptide recognition at the DRS. Structural analyses comparing phosphorylated and nonphosphorylated ERK2 may further clarify how activation‐associated conformational changes influence DRS accessibility and peptide binding [15].

The conventional strategy for kinase inhibition relies on small‐molecule inhibitors, often purine derivatives, that competitively bind the ATP‐binding site, thereby blocking ATP‐binding and suppressing kinase catalytic activity [16]. Despite the human kinome containing 538 kinases, the FDA has approved only about 70 kinase inhibitors for clinical use against approximately 30 targets [17]. ATP‐competitive inhibitors account for the vast majority of these compounds, whereas only a limited subset targets allosteric sites distinct from the ATP‐binding pocket [18, 19].

Although a plethora of ERK2 inhibitors have been developed, only a limited number are currently undergoing phase I/II clinical trials [20, 21, 22]. For instance, the reversible ATP‐competitive ERK1/2 inhibitor BVD‐523 is now in phase I and II clinical studies to treat malignant eye carcinoma, non‐Hodgkin lymphoma, and advanced pancreatic cancer [23]. Due to the fact that numerous kinases possess highly conserved ATP‐binding pockets, ATP‐competitive inhibition often suffers from limited selectivity, dose‐limiting toxicity, and the development of drug resistance [24, 25]. To overcome these limitations, peptide‐based ERK2 inhibitors have been developed that selectively target the DRS [26]. The DRS was selected because it represents a key docking interface for ERK2 substrates, regulators, and peptides containing the docking motif. Thus, DRS targeting may offer an alternative peptide‐based strategy for modulating ERK2‐mediated protein‐protein interactions beyond conventional ATP‐site inhibition. Notably, the kinase interaction motif of MAP kinase phosphatase 3, referred to as KIM‐MKP3 or KIM‐MAP3, has been structurally characterized in complex with ERK2 in the crystal structure deposited under PDB ID: 2FYS [27]. This peptide is derived from MAP kinase phosphatase 3 and binds to the ERK2 D‐recruitment site through interactions with the acidic patch and hydrophobic groove located in a noncatalytic region opposite to the kinase catalytic pocket. Therefore, KIM‐MAP3 was used in the present study as a structurally resolved DRS‐binding reference peptide, not as a functional inhibitory control.

Peptides constitute a distinctive class of bioactive compounds characterized by their high efficacy and therapeutic potential [28]. Of note, the strong and specific interactions of peptide‐based drugs with their protein targets underpin their remarkable selectivity and low toxicity [29]. Amphibian skin secretions involve a rich source of biologically active compounds, specifically peptides, which exhibit various functional properties [30]. The skin glands of frogs produce and secrete a remarkably diverse repertoire of hormones and neuropeptides resembling those found in mammals [31, 32]. Due to their intrinsic bioactivity, peptides extracted from frog skin secretions serve as effective anticancer therapies [33, 34, 35]. Specifically, several naturally occurring amphibian peptides and their analogs demonstrate selective cytotoxicity against tumor cells, illuminating their promise as anticancer drug candidates [36].

**FIGURE 2:**
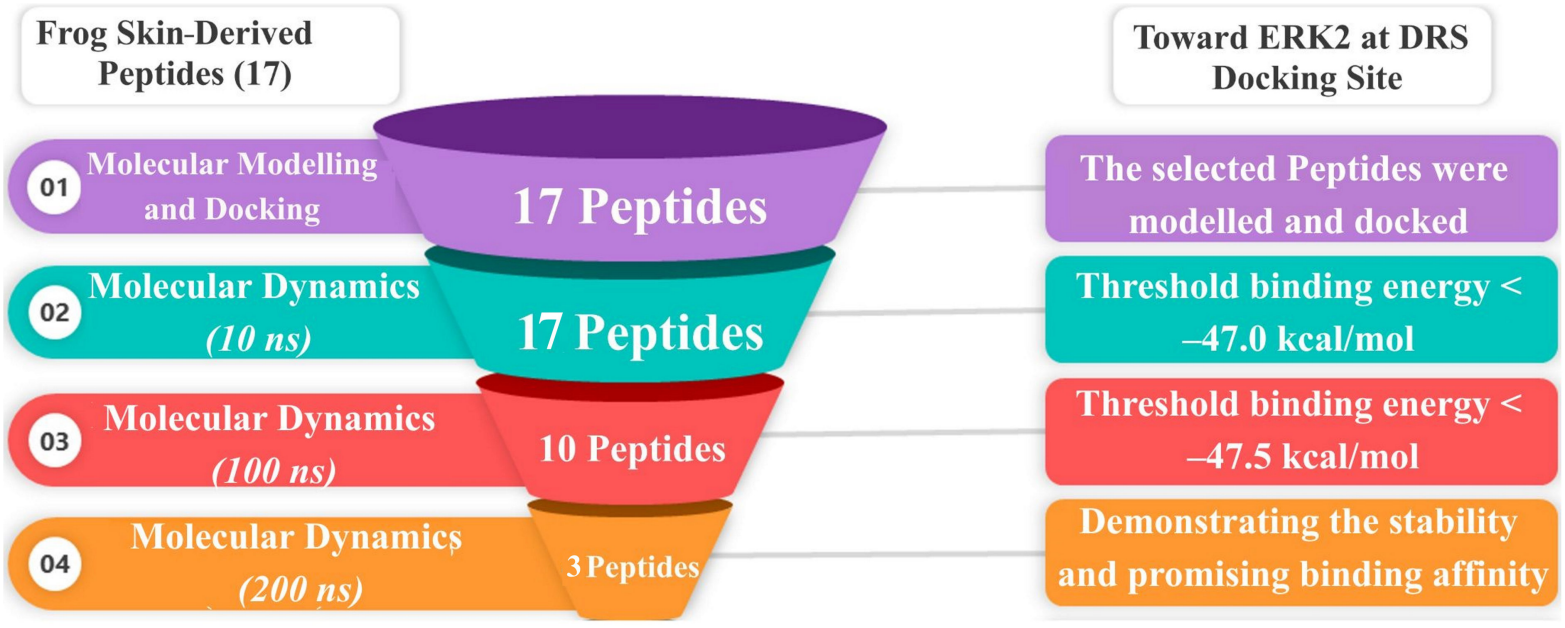
Schematic depiction of the employed in silico approaches for the frog skin‐derived peptides toward ERK2 DRS.

The present study was designed as a focused computational prioritization rather than an exhaustive survey of all known frog skin‐derived peptides. Accordingly, 17 peptides were selected from the literature based on their previously reported anticancer activity, providing a biologically relevant starting point for evaluating their potential interactions with the ERK2 DRS. The selected peptides were modeled and docked against ERK2 DRS. Subsequently, all investigated peptides complexed with ERK2 DRS were introduced to molecular dynamics simulations (MDS), along with binding energy computations employing the MM/GBSA approach. Structural and energetic analyses were then executed to evaluate the conformational stability and dynamic behavior of the identified peptides complexed with ERK2 DRS throughout 200 ns MDS. Moreover, the physicochemical properties of the most potent anticancer peptides were anticipated to provide deeper insights into their molecular features. The identified peptides can be regarded as promising ERK2 DRS‐binding candidates that require further validation through direct binding assays, ERK2 kinase activity assays, and functional in vitro/in vivo studies. Recent dry‐wet studies have highlighted the value of integrating computational screening with experimental validation [37]. The in silico workflow utilized for frog skin‐derived peptides toward ERK2 DRS is shown in Figure 2.

## 2 Computational Methodology

### 2.1 ERK2 Preparation

The 3D structure of the ERK2 complexed with KIM‐MKP3 peptide was retrieved from the Protein Data Bank (PDB accession code: 2FYS) [27]. For preparation purposes, all heteroatoms, including the cocrystallized peptide, ions, and water molecules, were removed. Missing nonterminal residues were modeled utilizing the Modeller software [38]. The PropKa program was applied to investigate the protonation states of the titratable amino acids [39]. Additionally, all missing H‐atoms were added to complete the structural preparation of ERK2.

### 2.2 Frog Skin‐Derived Peptide Preparation

A comprehensive literature survey identified 17 frog skin‐derived peptides with reported anticancer potential (Table 1). Peptides were selected according to the following criteria: (i) frog skin origin, (ii) availability of a complete primary amino acid sequence, and (iii) reported anticancer or cytotoxic activity in experimental studies. Peptides with incomplete sequences, ambiguous residue assignments, or insufficient biological annotation were not included. This selection strategy was adopted to focus the computational screening on peptides with prior biological relevance to cancer‐related applications. The 3D structures of these peptides were predicted using AlphaFold2 software, an advanced structure‐prediction tool known for achieving near‐experimental accuracy [52]. For each peptide, the highest confidence model was selected for subsequent computations. The protonation states of the modeled peptides were determined employing the PropKa program [39].

**TABLE 1:** Primary amino acid sequences and lengths of selected frog skin‐derived peptides with anticancer potential.

No.	Peptide name	Sequence length	Amino acid sequence	References
1	Alyteserin‐2	16	ILGKLLSTAAGLLSNL	[40]
2	Ascaphin‐8	19	GFKDLLKGAAKALVKTVLF	[41]
3	Aurein 1.2	13	GLFDIIKKIAESF	[42]
4	Aurein 3.1	17	GLFDIVKKIAGHIAGSI	[42]
5	Dermaseptin‐B2	33	GLWSKIKEVGKEAAKAAAKAAGKAALGAVSEAV	[31]
6	Dermaseptin‐B3	29	ALWKNMLKGIGKLAGQAALGAVKTLVGAE	[31]
7	Dermaseptin‐PD‐1	31	GMWSKIKETAMAAAKEAAKAAGKTISDMIKQ	[43]
8	Dermaseptin‐PD‐2	33	GMWSKIKNAGKAAAKAAAKAAGKAALDAVSEAI	[43]
9	Dermaseptin L1	32	GLWSKIKEAAKAAGKAALNAVTGLVNQGDQPS	[44]
10	Phylloseptin L1	18	LLGMIPLAISAISALSKL	[44]
11	Phylloseptin‐PBa	22	MAFLKKSLFLVLF(F/L)GLVSLSIC	[45]
12	Pentadactylin	25	GLLDTLKGAAKNVVGSLASKVMEKL	[46]
13	Hymenochirin‐1B	27	KLSPETKDNLKKVLKGAIKGAIVAKMV	[47]
14	Magainin‐2	23	GIGKFLHSAKKFGKAFVGEIMNS	[48]
15	XLAsp‐P1	5	DEDDD	[49]
16	Peptide XT‐7	17	GLLGPLLKIAAKVGSNLL	[50]
17	Esculentin‐2CHa	37	GFSSIFRGVAKFASKGLGKDLAKLGVDLVACKISKQC	[51]

### 2.3 Molecular Docking

Peptide‐ERK2 docking calculations were executed with the help of the open‐source HADDOCK2.4 platform to explore the interactions between the selected anticancer frog skin‐derived peptides and ERK2 DRS [53]. The docking process in HADDOCK2.4 software is driven by the anticipation of probable interface amino acids, referred to as ambiguous interaction restraints (AIRs), which guide the formation of the complex [54]. Herein, ERK2 active site residues—LYS112, THR116, TYR126, TYR129, THR157, GLN313, TYR314, and ASP319—were identified as proximal interaction parameters for docking calculations. On the other hand, all residues of the anticancer frog skin‐derived peptides were involved as active during the docking calculations.

### 2.4 MDS

All frog skin‐derived peptides complexed with ERK2 DRS were advanced for MDS utilizing AMBER20 software [55]. All MDS parameters and computational settings are documented in detail elsewhere [56, 57, 58, 59]. Briefly, the topology of ERK2 and the investigated peptides was described employing the AMBER force field 14SB [60]. Each peptide‐ERK2 complex was solvated in a truncated octahedral TIP3P water model, with a 12 Å distance from the ERK2 surface [61]. Subsequently, peptide‐ERK2 complexes were neutralized by inserting Na^+^/Cl^−^ counterions. For the prepared peptide‐ERK2 complexes, energy minimization was performed for 5000 cycles. The peptide‐ERK2 complexes were then progressively warmed up to 310 K over 50 ps, accompanied by an equilibration under the NPT ensemble for 10 ns. Production phases for the investigated peptide‐ERK2 complexes were conducted for 10, 100, and 200 ns, with coordinates captured every 10 ps. The GPU version of the PMEMD.CUDA inside the AMBER20 software was utilized to run MDS. All molecular interactions of peptides complexed with ERK2 DRS were visualized utilizing PyMOL software [62, 63].

### 2.5 Binding Energy Computation

The binding energy (Δ*G*_binding_) between ERK2 DRS and the investigated frog skin‐derived peptides was evaluated using the molecular mechanics/generalized Born surface area (MM/GBSA) approach [64]. The polar solvation participation was also calculated utilizing the generalized Born (GB) model of solvation as implemented by Onufriev et al. (igb = 2) [65]. The Δ*G*_binding_ was computed employing the subsequent equation:

$$\text{Δ}G_{\text{binding}}=G_{\text{Complex}}-\left(G_{\text{Peptide}}+G_{\text{Receptor}}\right)$$

where the energy symbol (*G*) was determined as

$$G=G_{\mathrm{GB}}+E_{\mathrm{vdW}}+E_{\mathrm{ele}}+G_{\mathrm{SA}}$$

*G*_SA_ and *G*_GB_ stand for the nonpolar and the electrostatic solvation free energies, respectively, while the *E*_ele_ and *E*_vdW_ indicate electrostatic and van der Waals energies, respectively. Due to high computational cost, the entropic participation was neglected [66, 67].

### 2.6 Physicochemical Properties

To gain a deeper insight into the physicochemical properties of potent peptides, various parameters were predicted utilizing the ProtParam server [68, 69, 70, 71, 72]. The estimated physicochemical properties included molecular weight (MW), half‐life, grand average of hydropathy (GRAVY), theoretical isoelectric point, instability index, and aliphatic index. The aliphatic index, representing the relative volume contributed by aliphatic side chains (LEU, ILE, VAL, and ALA), is commonly used as an indicator of peptide thermal stability [70]. GRAVY values were determined by averaging the hydropathy values of all residues in a peptide sequence, providing an assessment of the peptide’s overall hydrophilic or hydrophobic nature.

## 3 Results and Discussion

### 3.1 In Silico Protocol Assessment

**FIGURE 3:**
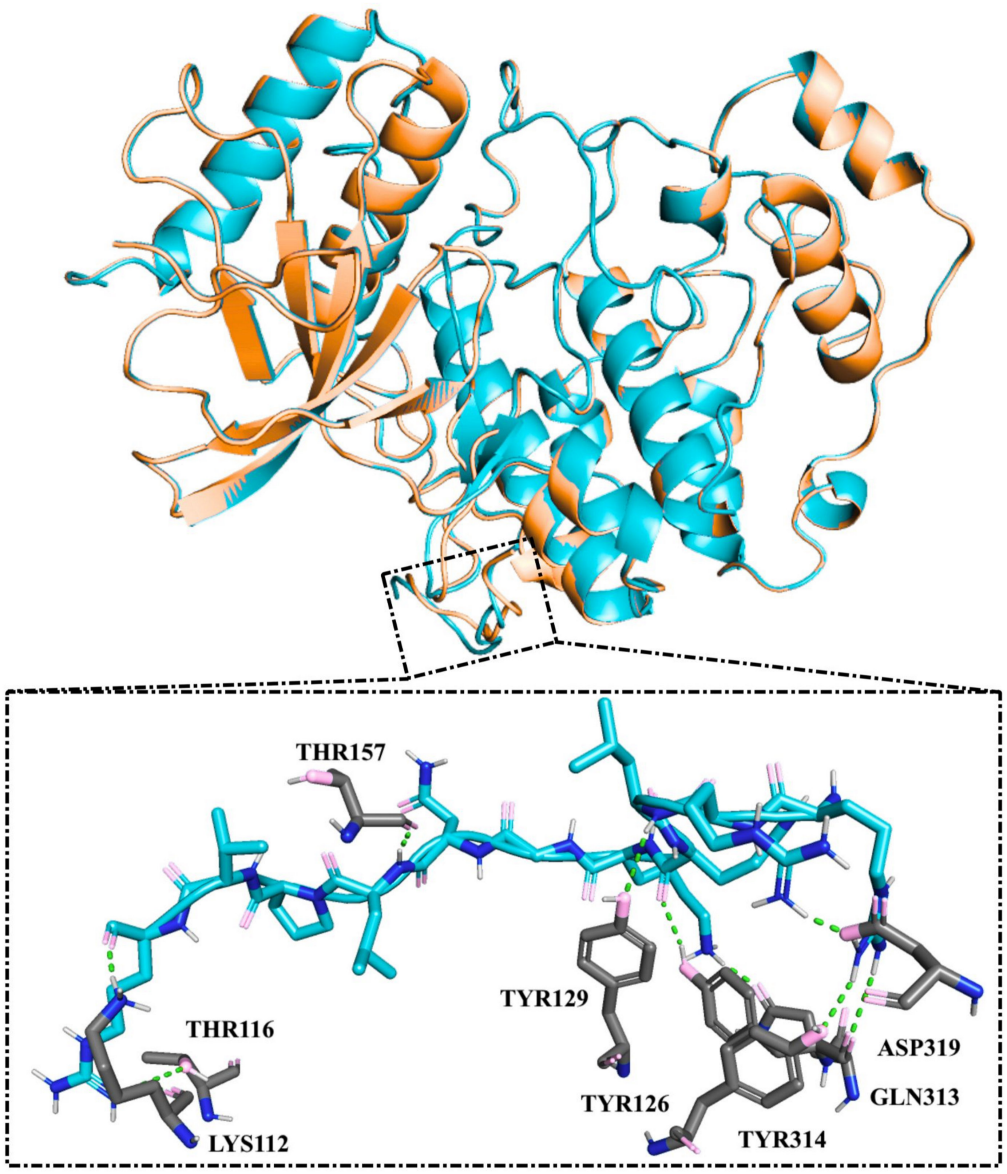
Superimposition of the experimentally resolved binding pose (cyan) with the predicted redocked poses (orange) of KIM‐MAP3 within the ERK2 DRS.

A validation process was executed to assess the reliability of the HADDOCK2.4 docking protocol for accurately predicting peptide binding modes at the ERK2 DRS. Initially, the cocrystalized KIM‐MAP3 peptide was prepared and redocked against ERK2 DRS (Figure 3). As shown in Figure 3, the predicted docking pose of the KIM‐MAP3 within the ERK2 at the DRS closely overlapped with the experimentally binding pose, displaying an RMSD with a value of 1.23 Å. As well, the predicted docking score of KIM‐MAP3 within the ERK2 at the DRS was found to be −322.1 kcal/mol. Inspecting the docking pose of KIM‐MAP3 disclosed that LYS112, THR116, THR157, TYR126, ASP319, TYR129, TYR314, and GLN313 of the ERK2 DRS formed H‐bonds with the ARG74, LEU71, ARG65, ARG64, GLN67, and LYS68 of the KIM‐MAP3 peptide, exhibiting bond lengths ranging from 1.5 to 2.7 Å. Consequently, these findings demonstrated that the HADDOCK2.4 software predicts the correct binding pose, confirming its reliability for subsequent docking calculations.

### 3.2 Molecular Recognition of ERK2 DRS by Frog Skin‐Derived Peptides

According to a comprehensive literature survey, 17 frog skin‐derived peptides with reported anticancer activity were collected and docked against the ERK2 DRS using HADDOCK2.4 software (Table S1). Based on data registered in Table S1, the investigated peptides demonstrated good docking scores with values spanning from −159.8 to −342.1 kcal/mol against ERK2 DRS. It is worth mentioning that docking scores provide an initial estimate of binding compatibility and should not be interpreted alone as evidence of biological inhibition. Therefore, docking was used only as a preliminary filtering step to identify peptides with favorable predicted interactions with the ERK2 DRS for subsequent MDS and MM/GBSA binding energy computations. Figure S1 illustrates the 3D depiction of the binding patterns for these peptides with the fundamental binding residues of ERK2 DRS. The majority of the investigated peptides exhibited similar docking poses, forming fundamental H‐bonds with TYR126, THR157, and ASP316 (Figure S1). The predicted docking scores and the potent polar contents for the top three potent peptides are listed in Table 2. Notably, these peptides were selected according to the estimated MM/GBSA binding energy from 200 ns MDS, as outlined below.

**TABLE 2:** Predicted docking scores and polar contents of the top three potent peptides and KIM‐MAP3 against ERK2 DRS.

Peptide name	Docking score, kcal/mol	Polar contents, Å
KIM‐MAP3	−322.1	LYS112‐ARG74 (1.6, 1.8 Å); THR116‐ARG74 (2.5 Å); THR157‐LEU71 (1.9 Å); TYR126‐LEU66 (2.7 Å); ASP319‐ARG65 (1.5 Å); TYR129‐ARG64 (2.3 Å), TYR314‐GLN67; TYR314‐ARG64 (2.1, 2.5 Å); GLN313‐LYS68 (1.7 Å)
Ascaphin‐8	−268.1	ASP160‐LYS11 (1.5 Å); GLU79‐LYS11 (1.9 Å); TYR129‐LYS7 (1.9 Å); ASP319‐LYS7 (2.5, 1.6 Å); TYR315‐LYS3 (1.6 Å); GLU312‐LYS3 (2.0 Å)
Dermaseptin‐B3	−243.8	GLU107‐LYS23 (1.6 Å), HIS123‐GLY11 (2.4 Å), ASP160‐LYS8 (1.6 Å), TYR126‐LYS8 (2.1 Å), GLU79‐LYS8 (2.2 Å), TYR129‐LYS4 (1.6 Å), ASP316‐ALA1 (1.7 Å)
Dermaseptin‐PD‐1	−236.4	THR157‐LYS23 (2.5 Å); HIS123‐ALA17 (2.2 Å); TYR123‐LYS15 (2.0 Å); GLU79‐LYS15 (2.3 Å); ASP316‐LYS7 (1.5 Å); ASP316‐SER4 (1.6, 2.7 Å); TYR315‐LYS7 (1.6 Å)

**FIGURE 4:**
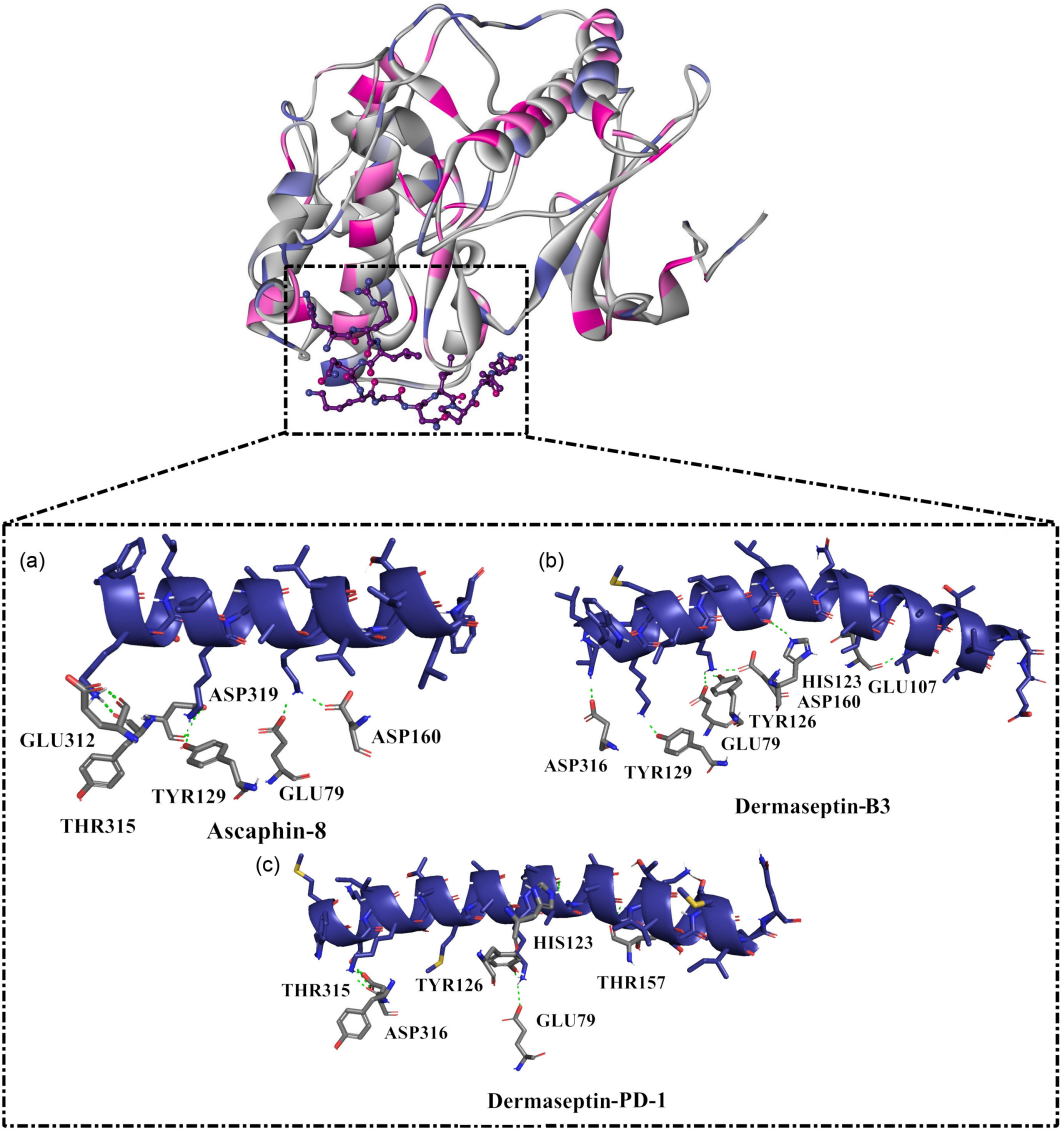
3D representations of the predicted docking modes of (a) ascaphin‐8, (b) dermaseptin‐B3, and (c) dermaseptin‐PD‐1 within ERK2 DRS.

Ascaphin‐8 is extracted from the skin secretions of Silurana tropicalis and Ascaphus truei, exhibiting considerable cytotoxicity toward HepG2 hepatocellular carcinoma cells [41]. Ascaphin‐8 displayed the lowest docking score of −268.1 kcal/mol against ERK2 DRS. Analyzing the predicted docking pose of ascaphin‐8 within the DRS of ERK2 revealed that ascaphin‐8 formed seven H‐bonds with ASP160, GLU79, TYR129, ASP319, TYR315, and GLU312 (Figure 4).

Dermaseptin‐B3, belonging to the dermaseptin peptide family, originates from the skin secretions of the South American tree frog *Phyllomedusa bicolour* and exhibits pronounced angiostatic and anticancer properties [31]. In particular, dermaseptin‐B3 inhibits the growth of the human prostate cancer cell line PC‐3 by more than 90% in vitro and suppresses the differentiation of bovine aortic endothelial cells [31]. Dermaseptin‐B3 unveiled the second‐lowest docking score of −243.8 kcal/mol toward ERK2 DRS. The promising docking score of dermaseptin‐B3 may be ascribed to its capacity to establish seven H‐bonds with GLU107, HIS123, ASP160, TYR126, GLU79, TYR129, and ASP316 with bond lengths ranging from 1.6 to 2.4 Å within the DRS of ERK2 (Figure 4).

Shi et al. reported the discovery of a new dermaseptin antimicrobial peptide, termed dermaseptin‐PD‐1, isolated from the skin and skin secretions of the phyllomedusine leaf frog (*Pachymedusa dacnicolor*) [43]. Dermaseptin‐PD‐1 effectively prohibits the proliferation of various human carcinoma cell lines, including the prostate cancer cell line PC‐3, the nonsmall cell lung cancer cell line H157, and the neuronal glioblastoma cell line U251MG, while exhibiting low hemolytic activity [43]. Dermaseptin‐PD‐1 displayed a favorable docking score of −236.4 kcal/mol toward ERK2 DRS and exhibited seven H‐bonds with proximal residues, involving THR157, HIS123, TYR123, GLU79, ASP316, and TYR315 (Figure 4).

### 3.3 MDS

**FIGURE 5:**
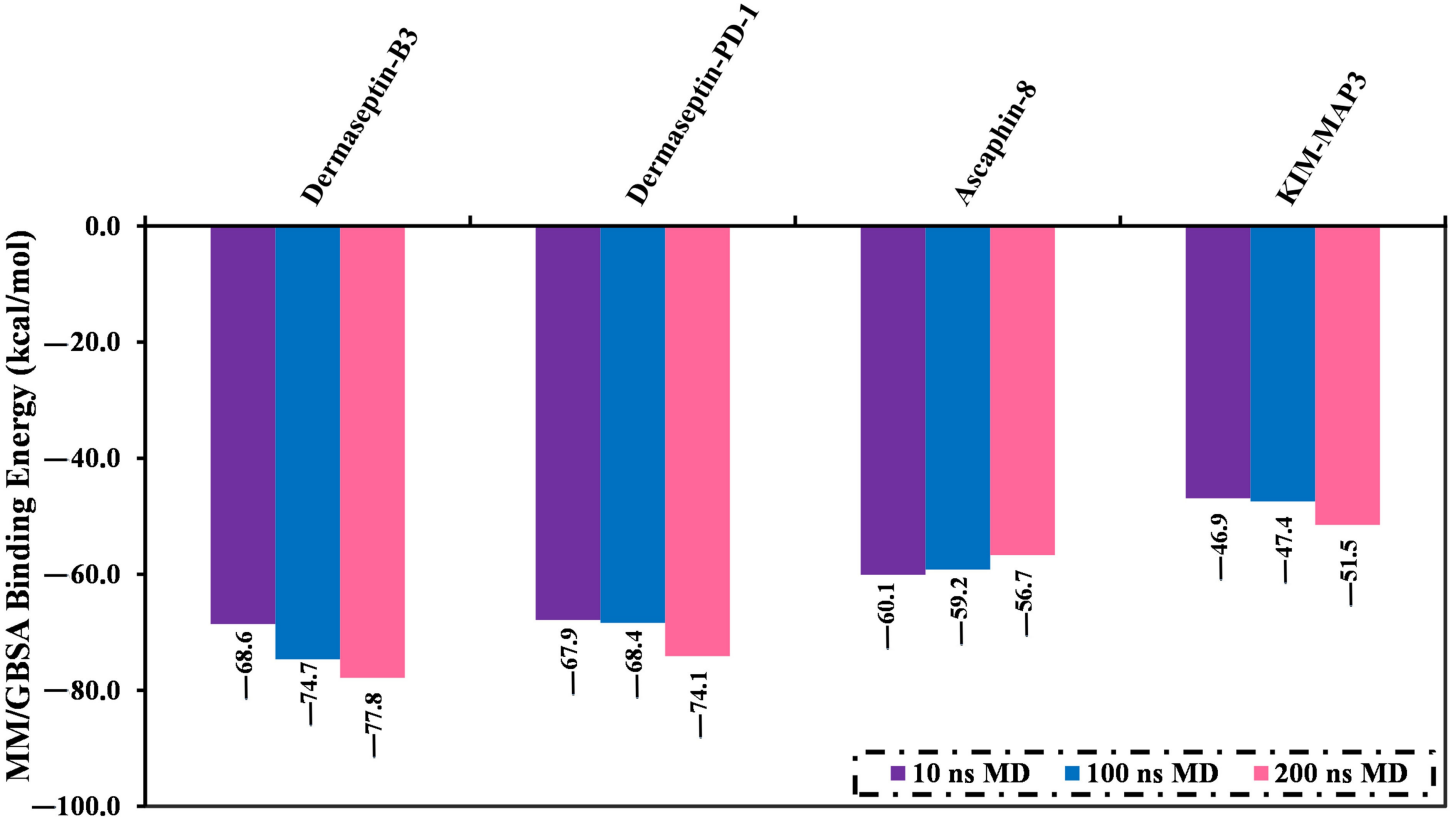
Binding energy evaluations of the identified peptides and KIM‐MAP3 complexed with ERK2 DRS over 10, 100, and 200 ns MDS.

MDS is a powerful computational technique utilized to inspect atomistic‐level interactions, the kinetic behavior of inhibitor‐receptor complexes, and conformational changes [65]. Accordingly, all investigated peptides complexed with ERK2 DRS were advanced for MDS over 10 ns, along with MM/GBSA binding energy (Δ*G*_binding_) evaluations (Table S2). Based on the data provided in Table S2, 10 peptides demonstrated binding affinities better than KIM‐MAP3 (calc. −46.9 kcal/mol), with Δ*G*_binding_ values spanning from −54.7 to −68.6 kcal/mol. To obtain more reliable results, these 10 peptides complexed with ERK2 DRS were further subjected to MDS over 100 ns, combined with binding energy calculations (Table S3). From Table S3, three peptides—namely, dermaseptin‐B3, dermaseptin‐PD‐1, and ascaphin‐8—exhibited superior binding affinities than the structurally resolved KIM‐MAP3 binding reference (calc. −47.4 kcal/mol). Therefore, these three promising peptides were advanced for an extended 200 ns MDS, accompanied by binding energy computations (Figure 5). Notably, there was no substantial variation in the assessed binding affinities for these peptides complexed with ERK2 DRS throughout 100 and 200 ns MDS. From Figure 5, dermaseptin‐B3, dermaseptin‐PD‐1, and ascaphin‐8 achieved less binding energies against ERK2 DRS with Δ*G*_binding_ values of −77.8, −74.1, and −56.7 kcal/mol, respectively, compared to KIM‐MAP3 (calc −51.5 kcal/mol).

**FIGURE 6:**
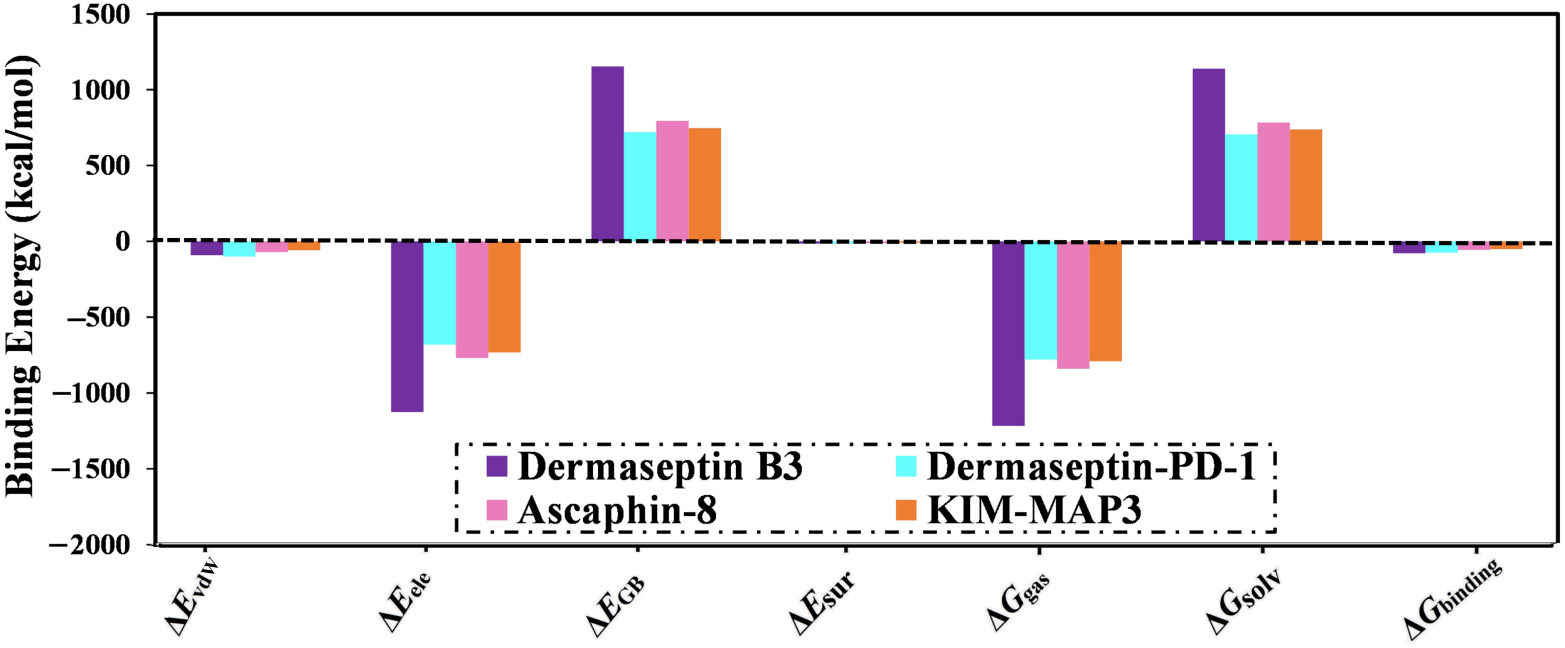
Binding energy decomposition of the identified peptides and KIM‐MAP3 complexed with ERK2 over a 200 ns MDS.

Additionally, the Δ*G*_binding_ was decomposed into its components to determine the dominant interaction forces participating in the binding of the identified peptides complexed with ERK2 DRS (Figure 6). As demonstrated in Figure 6, *E*_ele_ showed the major driving force in the binding of dermaseptin‐B3, dermaseptin‐PD‐1, ascaphin‐8, and KIM‐MAP3 complexed with ERK2 DRS, with mean values of −1125.6, −680.5, −768.1, and −731.7 kcal/mol, respectively. Besides, *E*_vdW_ manifested a favorable contribution in the binding of dermaseptin‐B3, dermaseptin‐PD‐1, ascaphin‐8, and KIM‐MAP3 complexed with ERK2 DRS, with average values of −91.4, −99.2, −71.4, and −58.1 kcal/mol, respectively. These results suggested that the strengthened electrostatic and van der Waals interactions played an important role in stabilizing the identified peptides inside the ERK2 DRS.

**FIGURE 7:**
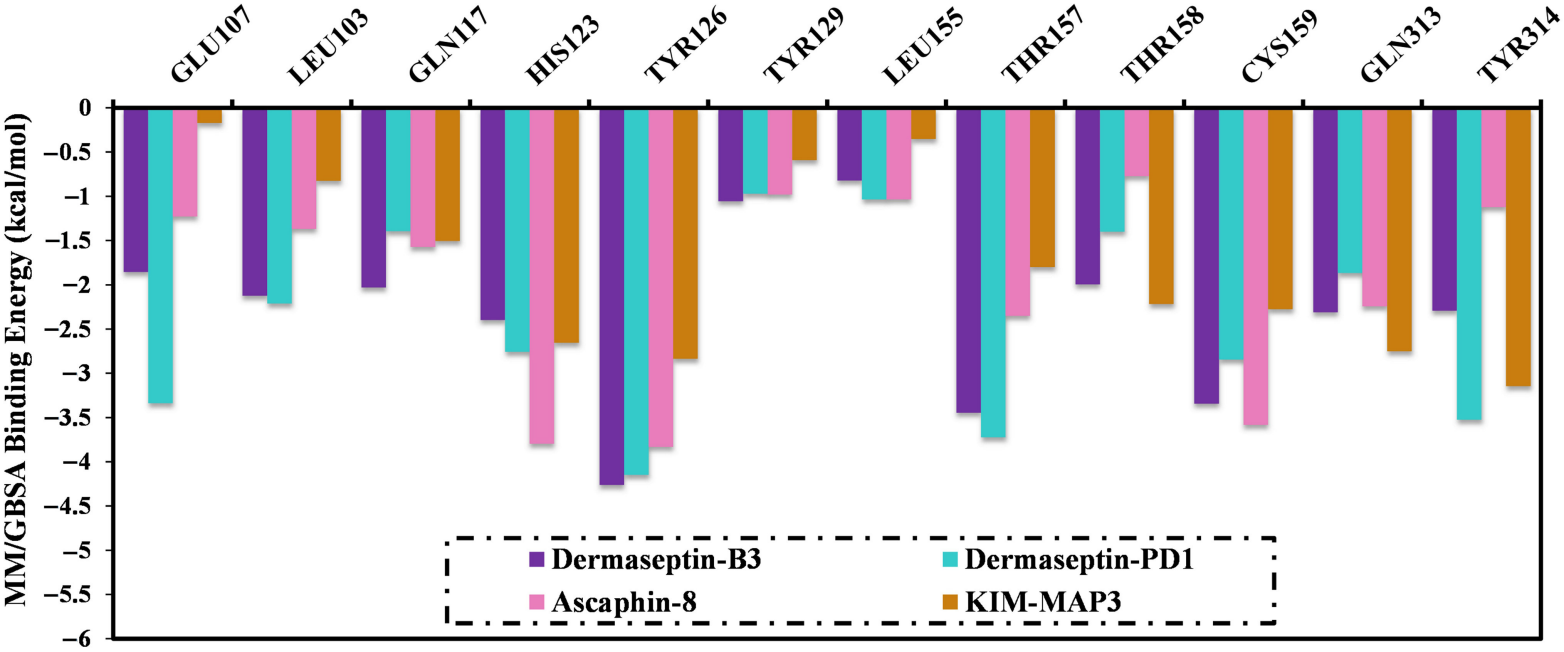
Per‐residue binding energy decomposition analysis of the identified peptides and KIM‐MAP3 complexed with ERK2 DRS throughout 200 ns MDS.

Furthermore, a per‐residue binding energy decomposition analysis was performed to identify the proximal residues of ERK2 DRS that contribute most significantly to interactions with the identified peptides. Notably, residues with energy contributions ≤−0.5 kcal/mol were included in the decomposition analysis (Figure 7). According to Figure 7, HIS123, TYR126, THR157, CYS159, and TYR314 were identified as the main interacting residues within the ERK2 DRS. Among them, TYR126 manifested the highest contribution to the binding of dermaseptin‐B3, dermaseptin‐PD‐1, and ascaphin‐8 complexed with ERK2 DRS, with Δ*G*_binding_ values of −4.3, −4.1, and −3.8 kcal/mol, respectively, compared to KIM‐MAP3 (calc. −2.8 kcal/mol). As well, THR157 revealed favorable participation in the binding of dermaseptin‐B3, dermaseptin‐PD‐1, ascaphin‐8, and KIM‐MAP3 complexed with ERK2 DRS, with Δ*G*_binding_ values of −3.4, −3.7, −2.4, and −1.8 kcal/mol, respectively.

### 3.4 Post‐MD Analyses

#### 3.4.1 Binding Energy per Trajectory

**FIGURE 8:**
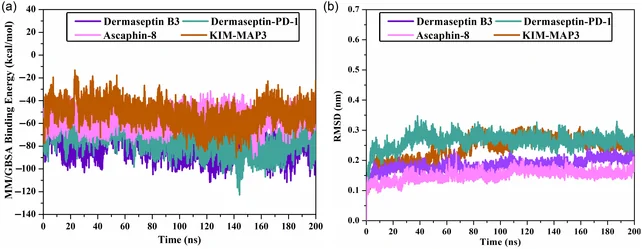
(a) Binding energy per trajectory and (b) RMSD for dermaseptin‐B3 (mauve), dermaseptin‐PD‐1 (cyan), ascaphin‐8 (pink), and KIM‐MAP3 (orange) complexed with ERK2 DRS over a 200 ns MDS.

The binding energy per trajectory was estimated for the identified peptides complexed with ERK2 DRS to monitor the energetic stability and conformational behavior of each complex over a 200 ns MDS (Figure 8a). Dermaseptin‐B3, dermaseptin‐PD‐1, and ascaphin‐8 complexed with ERK2 DRS manifested lower binding energy than the structurally resolved KIM‐MAP3 binding reference (calc. −51.5 kcal/mol), with values of −77.8, −74.1, and −56.7 kcal/mol, respectively. Collectively, the identified peptides demonstrated low atomic fluctuations over the 200 ns MDS, indicating high energetic constancy of peptide‐ERK2 complexes.

#### 3.4.2 Root‐Mean‐Square Deviation (RMSD)

RMSD is a standard metric used to assess the structural stability and conformational changes of inhibitor‐receptor complexes over MDS [73]. RMSD analysis was performed for the identified peptides and the KIM‐MAP3 complexed with ERK2 DRS over a 200 ns MDS (Figure 8b). As shown in Figure 8b, the identified peptides and KIM‐MAP3 exhibited an RMSD of 0.3 nm, indicating minimal structural deviation throughout the 200 ns MDS. These outcomes demonstrated that the identified peptides and KIM‐MAP3 maintained highly stable conformations relative to their initial structures, indicating strong and consistent binding to ERK2 DRS throughout the simulation course.

#### 3.4.3 Root‐Mean‐Square Fluctuation (RMSF)

**FIGURE 9:**
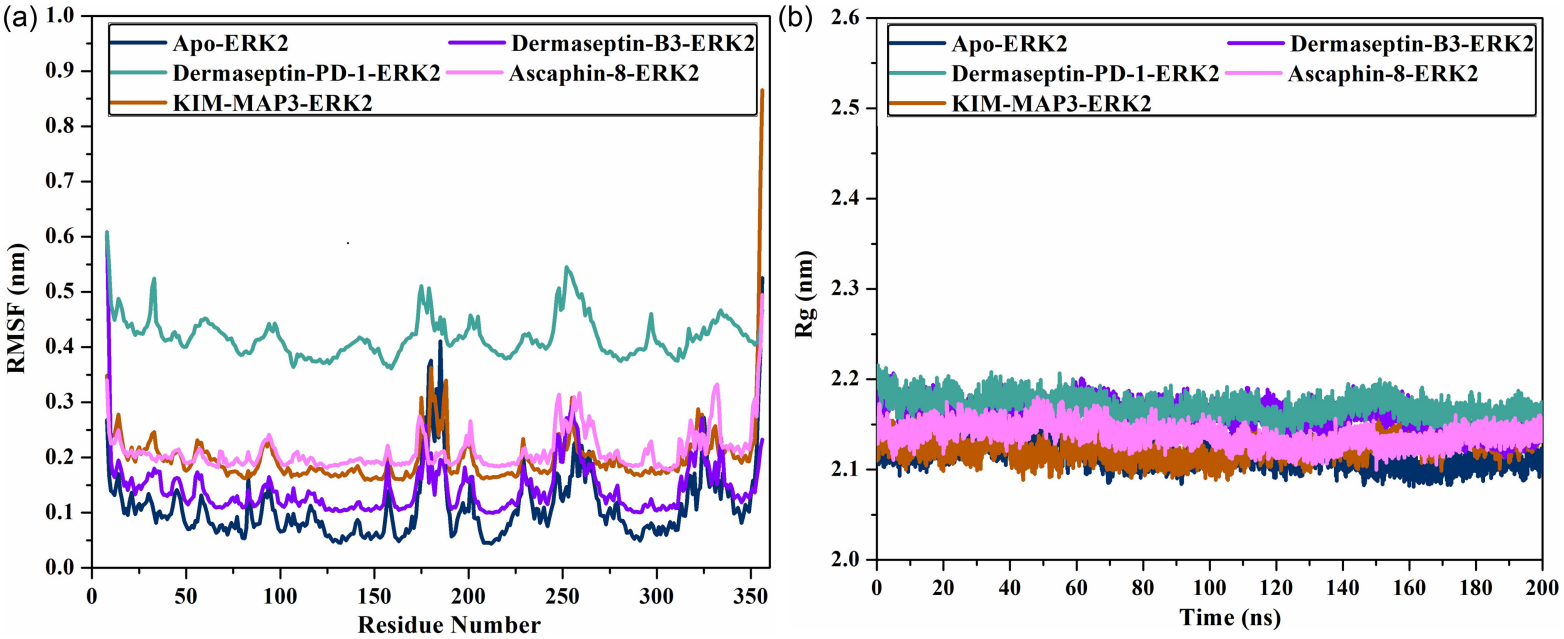
(a) RMSF and (b) Rg for apo‐ERK2 (navy), dermaseptin‐B3 (mauve), dermaseptin‐PD‐1 (cyan), ascaphin‐8 (pink), and KIM‐MAP3 (orange) complexed with ERK2 DRS over a 200 ns MDS.

Moreover, RMSF analysis was executed on the identified peptides complexed with ERK2 DRS to estimate the flexibility of residues relative to apo‐ERK2 over a 200 ns MDS (Figure 9a). According to Figure 9a, apo‐ERK2 and dermaseptin‐B3, dermaseptin‐PD‐1, ascaphin‐8, and KIM‐MAP3 complexed with ERK2 DRS disclosed comparable RMSF profiles, with average values of 0.1, 0.1, 0.4, 0.2, and 0.2 nm, respectively. Generally, the peptide‐ERK2 complexes exhibited lower atomic fluctuations than the apo‐ERK2 form, indicating limited structural flexibility and supporting the high stability of the peptide‐ERK2 complexes during the 200 ns MDS.

#### 3.4.4 Radius of Gyration (Rg)

The Rg analysis was also carried out for the identified peptides complexed with ERK2 DRS and apo‐ERK2 to assess their compactness and overall structural stability over a 200 ns MDS (Figure 9b). As displayed in Figure 9b, the peptide‐ERK2 complexes and the apo‐ERK2 demonstrated similar Rg profiles, with an average Rg value of 2.1 nm. These outcomes indicated that binding the identified peptides did not alter the global folding or compactness of the ERK2 backbone, confirming the high structural steady of the peptide‐ERK2 complexes throughout the simulation course.

#### 3.4.5 H‐Bond Analysis

**FIGURE 10:**
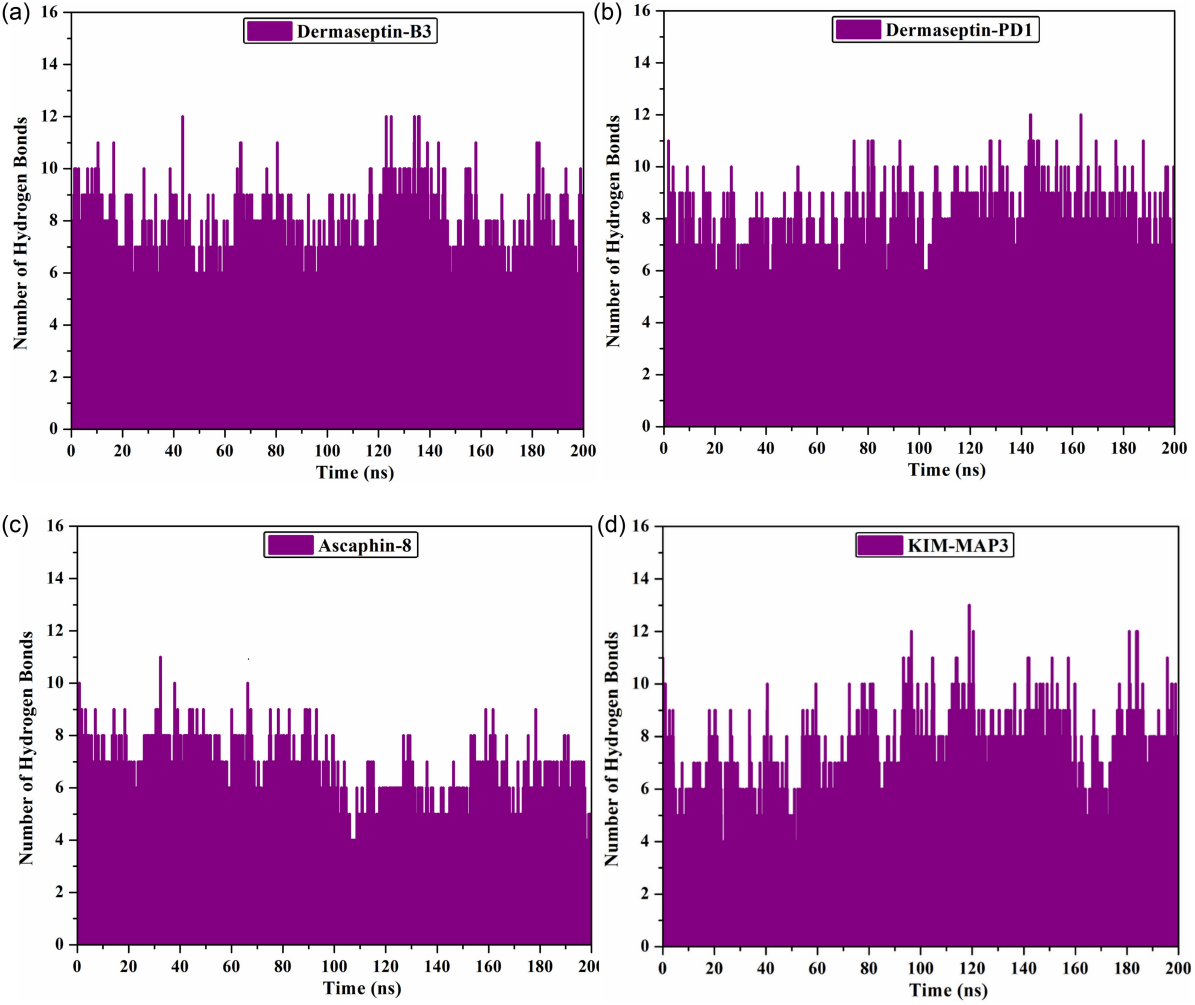
H‐bond number of (a) dermaseptin‐B3, (b) dermaseptin‐PD‐1, (c) ascaphin‐8, and (d) KIM‐MAP3 complexed with ERK2 DRS over a 200 ns MDS.

The number of H‐bonds formed within ligand‐protein complexes acts as a key indicator of complex stability and binding affinity [74]. Accordingly, the H‐bond analysis was employed to assess the interactions between the investigated peptides and ERK2 DRS over a 200 ns MDS (Figure 10). As represented in Figure 10, dermaseptin‐B3, dermaseptin‐PD‐1, ascaphin‐8, and KIM‐MAP3 established eight, nine, seven, and seven H‐bonds with ERK2 over a 200 ns MDS. The consistent H‐bond formation observed for these peptides throughout the simulation time reflects their stable binding affinity toward ERK2 DRS. Although the MDS and post‐MD analyses supported stable binding of the selected peptides to the ERK2 DRS, these computational results did not directly confirm ERK2 inhibition. Functional inhibition would require experimental validation using kinase activity assays, binding assays, and cell‐based studies. Accordingly, the identified peptides were discussed here as promising ERK2 DRS‐binding candidates rather than experimentally confirmed ERK2 inhibitors.

### 3.5 Physicochemical Features

The physicochemical profiles of the identified peptides and KIM‐MAP3 were assessed employing the Expasy ProtParam tool, and the results are compiled in Table 3. The computed MWs of the dermaseptin‐B3, dermaseptin‐PD‐1, ascaphin‐8, and KIM‐MAP3 were found to be 2909.53, 3266.88, 2019.50, and 1336.61 Da, respectively. Peptide stability in vivo was predicted on the basis of the instability index, where values below 40 indicate stable peptides [75]. The instability indices of dermaseptin‐B3, dermaseptin‐PD‐1, and ascaphin‐8 were −6.59, 10.34, and −13.36, respectively, indicating strong stability profiles. However, KIM‐MAP3 showed an instability index of 110.38, indicating lower stability. All investigated peptides unveiled a high aliphatic index, with values of 121.38, 63.55, 128.42, and 97.27 for dermaseptin‐B3, dermaseptin‐PD‐1, ascaphin‐8, and KIM‐MAP3, respectively. A higher aliphatic index demonstrates enhanced thermal stability owing to the presence of aliphatic side chains [76, 77]. The GRAVY values further revealed that dermaseptin‐PD‐1 (−0.274) and KIM‐MAP3 (−1.327) were hydrophilic, whereas dermaseptin‐B3 (0.514) and ascaphin‐8 (0.737) were hydrophobic, suggesting good solubility and membrane interaction potentials.

**TABLE 3:** Anticipated physicochemical properties of the identified anticancer peptides from frog skin and KIM‐MAP3.

Peptides ID	MW	Theoretical isoelectric point	Estimated half‐life	Instability index	Aliphatic index	GRAVY
KIM‐MAP3	1336.61	12.30	1 h (mammalian reticulocytes, in vitro)	110.38	97.27	−1.327
			2 min (yeast, in vivo)			
			2 min (*Escherichia coli*, in vivo)			
Dermaseptin‐B3	2909.53	10.00	4.4 h (mammalian reticulocytes, in vitro)	−6.59	121.38	0.514
			>20 h (yeast, in vivo)			
			>10 h (*Escherichia coli*, in vivo)			
Dermaseptin‐PD‐1	3266.88	9.70	30 h (mammalian reticulocytes, in vitro)	10.34	63.55	−0.274
			>20 h (yeast, in vivo)			
			>10 h (*Escherichia coli*, in vivo)			
Ascaphin‐8	2019.50	10.0	30 h (mammalian reticulocytes, in vitro)	−13.36	128.42	0.737
			>20 h (yeast, in vivo)			
			>10 h (*Escherichia coli*, in vivo)			

## 4 Conclusion

ERK2 plays a vital function in promoting cell survival and proliferation by phosphorylating various substrates. ERK2 is a critical and druggable therapeutic target because of its role in dysregulated cell proliferation and cancer progression, underscoring the need for selective ERK2 inhibitors in the treatment of aggressive malignancies. Peptides are promising therapeutic agents with strong biological activity, and frog skin‐derived peptides have shown notable anticancer potential. In this study, 17 curated frog skin‐derived peptides with anticancer potential were modeled and submitted to molecular docking calculations against ERK2 DRS. Subsequently, all investigated peptides complexed with ERK2 DRS were introduced to MDS, combined with binding energy estimations. Based on MM/GBSA//200 ns MDS, dermaseptin‐B3, dermaseptin‐PD‐1, and ascaphin‐8 exhibited lower binding energies than KIM‐MAP3 (calc. −51.5 kcal/mol), with values of −77.8, −74.1, and −56.7 kcal/mol, respectively. Post‐MD analyses further assured the significant stability of the identified peptides complexed with ERK2 DRS over a 200 ns MDS. Finally, the physicochemical properties revealed that the identified peptides exhibited favorable functional potential. Overall, this study identifies dermaseptin‐B3, dermaseptin‐PD‐1, and ascaphin‐8 as promising frog skin‐derived peptides with favorable binding to the ERK2 DRS. Therefore, the identified peptides should be regarded as candidate ERK2 DRS binders rather than experimentally confirmed ERK2 inhibitors. Further in vitro and in vivo investigations, including direct ERK2 binding assays, kinase inhibition assays, and cancer cell‐based validation, are required to confirm their biological activity and therapeutic relevance. In addition, comparative binding analyses against other ERK2 functional regions will be necessary to assess the DRS selectivity of these peptides.

## Author Contributions

**Nahlah Makki Almansour:** conceptualization, methodology, software, resources, project administration, visualization, validation, writing – review and editing. **Mahmoud M. H. Abdelhamid:** data curation, formal analysis, investigation, visualization, writing – original draft. **Zahraa Alali:** investigation, visualization, writing – review and editing. **Mahmoud A. A. Ibrahim:** conceptualization, methodology, software, resources, project administration, supervision, writing ‍–‍ review and editing.

## Funding

This work was supported by the Deputyship for Research & Innovation, Ministry of Education, in Saudi Arabia (0037‐1446‐S).

## Conflicts of Interest

The authors declare no conflicts of interest.

## Supporting information

Supplementary Material

## Data Availability

The data that support the findings of this study are available in the Supporting Information of this article.
