# Supplementary material for: Targeting the ERK2 Signaling Pathway With Frog Skin‐Derived Bioactive Peptides: Insights From Molecular Modeling, Molecular Dynamics, and Physicochemical Features
Source: ChemistryOpen. 2026 Aug 24;15(9):e70295. doi: 10.1002/open.70295 (PMC13504238; doi:10.1002/open.70295)
Supplement: Supplementary file 1 [file OPEN-15-e70295-s001.pdf]

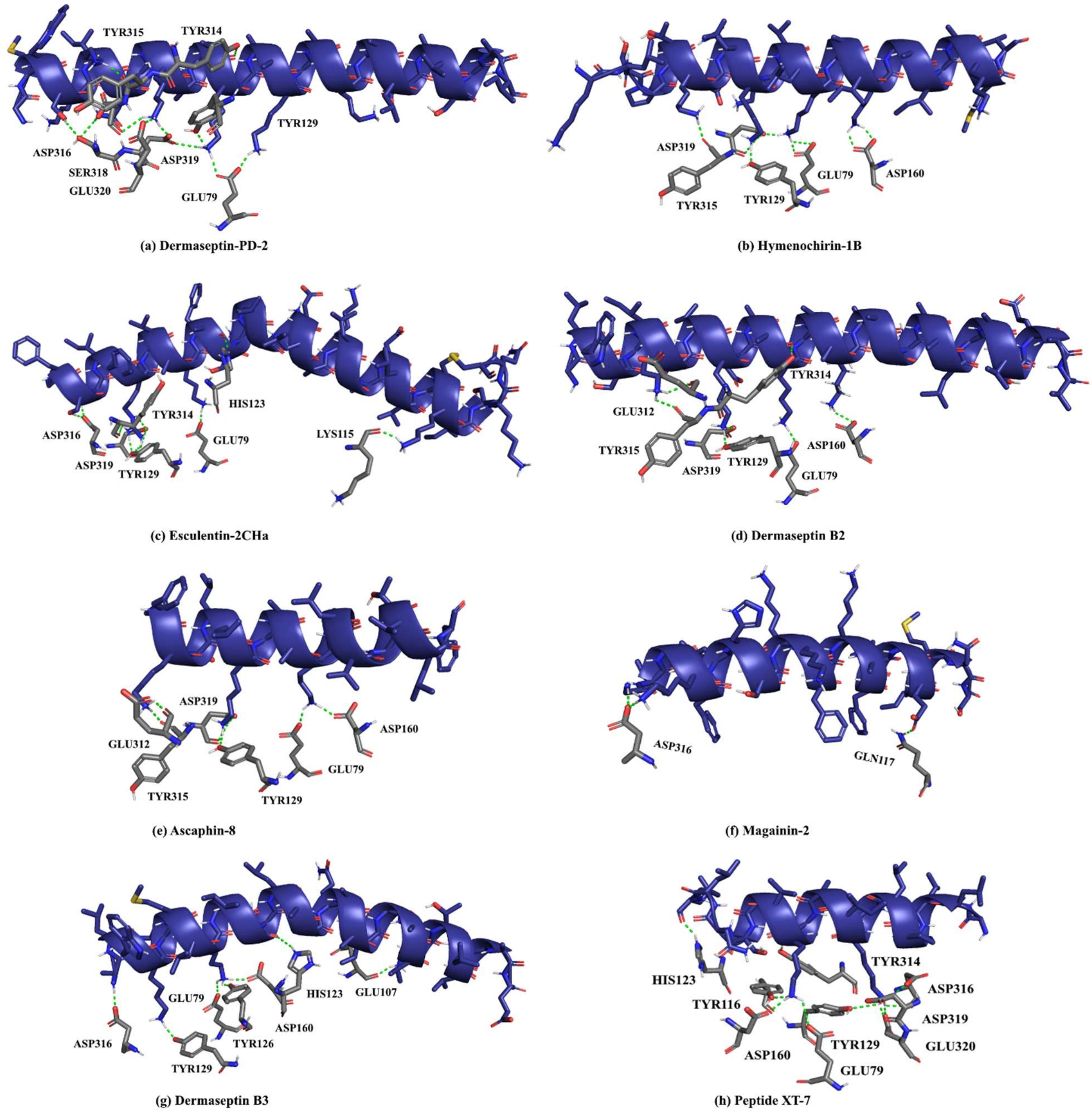

Figure S1. 3D representation of the predicted docking poses of the investigated peptides within the ERK2 DRS.

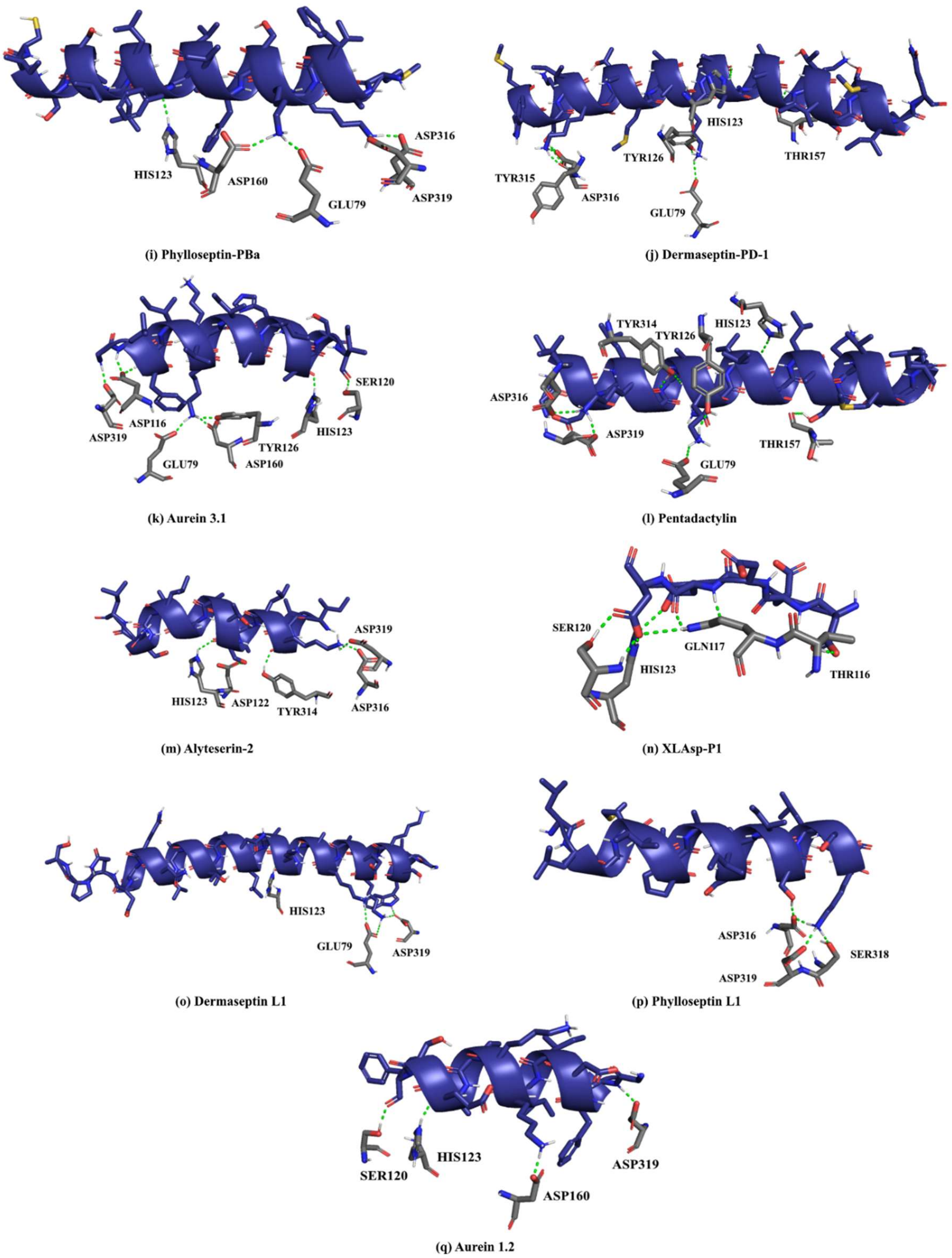

Figure S1. *Continued.*

Table S1. The computed docking scores of the investigated 17 frog skin-derived peptides towards ERK2 DRS.

**Peptide Name**	**Docking Score (kcal/mol)**	**Peptide Name**	**Docking Score (kcal/mol)**
KIM-MAP3	–322.1	Phylloseptin-PBa	–238.4
Dermaseptin-PD-2	–342.4	Dermaseptin-PD-1	–236.4
Hymenochirin-1B	–308.2	Aurein 3.1	–218.2
Esculentin-2CHa	–300.2	Pentadactylin	–217.8
Dermaseptin-B2	–279.0	Alyteserin-2	–198.3
Ascaphin-8	–268.1	XLAsp-P1	–184.9
Magainin-2	–259.1	Dermaseptin L1	–183.2
Dermaseptin-B3	–243.8	Phylloseptin L1	–176.7
Peptide XT-7	–241.0	Aurein 1.2	–159.8

Table S2. Calculated binding energies of the investigated 17 frog skin-derived peptides towards ERK2 DRS over 10 ns MD simulations.

Peptide Name	MM/GBSA Binding Energy (kcal/mol)	Peptide Name	MM/GBSA Binding Energy (kcal/mol)
KIM-MAP3	–46.9	Dermaseptin-B2	–52.5
Pentadactylin	–71.3	Esculentin-2CHa	–51.9
Dermaseptin-B3	–68.6	Aurein 3.1	–36.6
Dermaseptin-PD-2	–68.3	Peptide XT-7	–35.4
Dermaseptin-PD-1	–67.9	Alyteserin-2	–35.1
Ascaphin-8	–60.1	XLAsp-P1	–34.9
Hymenochirin-1B	–57.2	Dermaseptin L1	–33.7
Magainin-2	–54.8	Phylloseptin L1	–32.3
Phylloseptin-PBa	–54.7	Aurein 1.2	–30.1

Table S3. Calculated binding energies of the top ten potent peptides derived from frog skin towards ERK2 DRS over 100 ns MD simulations.

Peptide Name	MM/GBSA Binding Energy (kcal/mol)	Peptide Name	MM/GBSA Binding Energy (kcal/mol)
KIM-MAP3	–47.7	Hymenochirin-1B	–46.1
Dermaseptin-B3	–74.7	Pentadactylin	–45.3
Dermaseptin-PD-1	–68.4	Dermaseptin-PD-2	–44.0
Ascaphin-8	–59.2	Phylloseptin-PBa	–43.3
Magainin-2	–47.2	Dermaseptin-B2	–42.7
Esculentin-2CHa	–46.4		
